# Academic motivation in association with mental health and quality of life among medical and health science students: a survey in Vietnam

**DOI:** 10.1186/s12909-025-07368-4

**Published:** 2025-07-01

**Authors:** Ngoc Le Mai Duong, Viet Ha Nguyen, Minh Tri Ngo, Dai Minh Le, Tien Dat Nguyen, Thi Thu Trang Nguyen, Huu Tu Nguyen, Bao Giang Kim, Thanh Tung Pham

**Affiliations:** 1 Doctor of Medicine, Hanoi Medical University, Hanoi, Vietnam; 2 College of Health Sciences, VinUniversity, Hanoi, Vietnam; 3 Center for Assessment and Quality Assurance, Hanoi Medical University, Hanoi, Vietnam; 4 Department of Anesthesiology, Hanoi Medical University, Hanoi, Vietnam; 5 School of Preventive Medicine and Public Health, Hanoi Medical University, Hanoi, Vietnam; 6 Department of Epidemiology, Harvard T.H. Chan School of Public Health, Boston, USA; 7 Research Advancement Consortium in Health, Hanoi, Vietnam

**Keywords:** Academic motivation, Medical students, Medical education, Quality of life, Anxiety, Depression

## Abstract

**Introduction:**

Academic motivation is crucial in education, it was also found to be associated with various health-relating factors, but it has yet to be well-applied in the admission process of most Vietnamese medical universities. This study aimed to describe students’ academic motivation and investigate the association between it and depression, anxiety, and quality of life at Hanoi Medical University.

**Methods:**

A cross-sectional study was conducted on first and final year students of doctor and bachelor programs from October to November 2018 via a computer-based questionnaire. Academic motivation was assessed using the AMS-C 28 scale. The relationships between academic motivation and anxiety (screened by GAD-7), depression (screened by PHQ-9), and quality of life (assessed by SF-12) were analyzed using modified Poisson regression with robust error variances and linear regression models.

**Results:**

Among 1723 participants (85.1% response rate), 57.1% were female, 58.4% were first-year, and 75.7% were in doctor programs. The percentage of non-self-determined students was 37.9% (95%CI: 35.3 − 40.5%). The proportion of self-determined students was significantly higher among female compared to male participants (65.1% vs. 58.1%; *p* < 0.001), among first-year compared to final year ones (68.9% vs. 53.0%; *p* < 0.001), and among those in bachelors compared to doctor programs (68.7% vs. 60.2%, *p* < 0.001). Our Poisson regression and linear regression analysis showed a statistically significant association between academic motivation and anxiety disorder (PR = 1.44, 95%CI: 1.03–2.01); depression (PR = 1.62; 95%CI: 1.27–2.06); and quality of life: physical (Coef = -1.47, 95%CI: -2.19 - -0.74) and mental health components (Coef = -2.45, 95%CI: -3.34 - -1.55).

**Conclusions:**

Female, first-year, and bachelor of health sciences students tended to be more self-determined. More self-determined students were found to have a lower prevalence of depression, anxiety, and higher quality of life. Academic motivation should be further studied to be considered as an admission criterion during the selection process of medical schools to improve the quality of medical education in the future.

**Supplementary Information:**

The online version contains supplementary material available at 10.1186/s12909-025-07368-4.

## Introduction

Around the world, the process of selecting candidates for medical school admission is highly competitive. Potential candidates must demonstrate not only high academic records but also a genuine passion for medicine; as medical schools try to apply selection procedures that will enable them to select the most motivated students for their training program [1]. The admission process, therefore, varies between countries. The United States medical schools, for example, often require candidates to submit a personal statement besides a solid academic record and undergo rounds of interviews so that admission committees can assess their commitment and passion for medicine, to gain better insights into candidates’ aspirations and reasons behind their career choice [2]. On the other hand, Asian countries like China, Japan, and Vietnam emphasize test scores as the most critical criterion for medical admission [3–6]. In Vietnam, society paid little attention to the academic motivation of college students, and the selection criteria for most medical and pharmaceutical schools in Vietnam are still primarily based on academic achievement, as demonstrated by grades in National Entrance Examination, and still haven’t include academic motivation [4]. Utilizing test results is the most popular method for admission to medical universities because it enables institutions to choose students for the upcoming academic year based on their standards. However, there is a growing trend towards research about using other necessary characteristics such as academic motivation as admission factors since pure academic ability is no longer sufficient for the selection of candidates in medical school [7], given that an admission based solely on academic records would not fully capture other qualities needed for healthcare professionals [1, 6, 8].

Academic motivation is defined by a student’s desire demonstrated by their approach, perseverance, and level of interest in educational topics, particularly when their proficiency is compared to a standard of performance or excellence [9]. According to theory by Ryan and Deci [10], there are two types of motivation including intrinsic motivation - refers to engaging in a task of activity because of the inherent satisfaction it brings; and extrinsic motivation - implies when an activity is performed with the purpose to attain an outcome that is separable from the activity itself. To fully understand human behavior, Deci and Ryan posited amotivation - refers to the lack of perception of any connection between outcomes and their own activities [10–12] - to the motivation construct. Academic motivation is an important foundation of learning and reflects the direction of scholastic behavior [13]. Academic motivation has a favorable effect on academic achievement, whereas intrinsic and extrinsic motivation have been demonstrated to predict self-efficacy positively [14]. To excel in the field of medicine, high levels of motivation are necessary [15] since medical students who exhibit higher levels of academic motivation have been found to have higher average marks in both the pre-clinical and clinical phases of their study [16]. Besides academic performance, academic motivation may also have a certain effect on students’ well-being in medical school [3]. Therefore, a better understanding of the academic motivation in medical education is crucial, as students in this environment study with high intensity, whilst having to balance clinical duties and studies, in order to develop competencies required for medical practice [17].

Academic motivation was also found to be negatively associated with depression and stress in medical students [18, 19], and motivation was associated with the quality of life of medical and nursing students in previous research [20–22]. As medical training requires students to handle an enormous amount of physical and mental workload [23], most medical students suffer more distress than the general population [24, 25]. Recent meta-analysis studies revealed that one–third of medical students globally suffered from depression and anxiety [24, 26]. Thus, there is a need to study academic motivation and health-relating factors to enhance students’ learning productivity and quality of life. However, the differences in culture, social values, the admission process and the training curriculum [4], would likely make academic motivation varies between different contexts, it would be necessary to investigate the medical students’ academic motivation and its association with health outcomes in Vietnam. Therefore, this study aimed to describe the academic motivation of students and investigate the association between academic motivation and depression, anxiety, and students’ quality of life at Hanoi Medical University, in Vietnam during the academic year 2018–2019.

## Method

### Study setting, study design, and sampling

A cross-sectional study was conducted at Hanoi Medical University (HMU), which is known as one of the largest public higher medical institutions in Vietnam. HMU offers two different undergraduate training tracks: doctor lasting 6 years and bachelor for 4 years [4]. All students in the first and final years (the 4th year for Bachelor of Health Sciences programs and the 6th year for doctor programs) were invited to join our study. First-year students often face significant changes as they move from their high school experience to the new university environment [27]. Final-year students, by contrast, are approaching the end of their training and facing important career transitions [28]. Therefore, we focused on first- and final-year doctoral and bachelor students on their academic motivation and health-related factors at key points in their medical education.

According to circular number 03/2015/TT-BGDĐT by the Ministry of Education and Training (MOET) - Government of the Socialist Republic of Vietnam, and the Admission Plan of HMU 2018 number 357/ĐHYHN-ĐTĐH, at the time we conducted the research, Math, Chemistry, and Biology are the three subjects required for candidates seeking admission to medical school [29, 30]. The qualified students will be selected based on their score rankings and the number of available training positions for each institution; motivation was not a criterion for admission. Besides, students who get certain International Excellent Student Awards, National Excellent Student Awards, and Technical Science Awards would be considered for direct admission to HMU, with the number of candidates being admitted directly not exceeding 15% for each major [30].

In Vietnam, evaluating students based on the academic motivation when applying to medical universities is still not widely practiced. Utilizing test results is the most popular method for admission to medical universities in Vietnam because it enables institutions to choose students for the upcoming academic year based on their standards. At the time we conducted the research, Math, Chemistry, and Biology are the three subjects required for candidates seeking admission to medical school [29, 30]. The qualified students will be selected based on their score rankings and the number of available training positions for each institution; motivation was not a criterion for admission. Besides, students who get certain International Excellent Student Awards, National Excellent Student Awards, and Technical Science Awards would be considered for direct admission to HMU, with the number of candidates being admitted directly not exceeding 15% for each major [30].

Data was collected at the beginning of the first semester of the academic year 2018–2019 (from October to November 2018) via a computer-based questionnaire integrated into the tablets, after students finished the test at the Center of Assessment and Quality Assurance – Hanoi Medical University, with the representative of research team members to guide students in completing the electronic questionnaire. Each student was able to answer once, and we tracked responses using a single confidential code that linked to each student to prevent duplication. Additionally, we used the STATA command and the corresponding packages to check for any duplicate responses from the same confidential code and found no duplicate responses. Students who are on leave of absence or not in the first or final year of their study will be excluded. In the end, among 2025 invited students, 1723 students accepted to participate in this study, accounting for 85.1% (eMethods and eTable 1 – Supplemental 1).

### Instruments

The instruments included questions on socio-demographic information (genders, ethnic groups, perceived financial status, type of housemate, marital status), academic factors (academic year, academic majors), the Generalized Anxiety Disorder Questionnaire (GAD-7) to screen for anxiety, the Patient Health Questionnaire (PHQ-9) to screen for depressive symptoms, the Physical and Mental Health Summary Scales (SF-12) to evaluate the quality of life, and the Academic Motivation Scale (AMS-C 28) used for assessing students’ academic motivation.

### The generalized anxiety disorder

The Generalized Anxiety Disorder (GAD-7) developed by Spitzer et al. consisting of 7 items is an efficient tool to screen and assess the severity of anxiety disorder in research and practices [31]. With Cronbach α = 0.92, the internal consistency of the GAD-7 was excellent (Cronbach = 0.92) and test-retest reliability was also good (intraclass correlation = 0.83) [31]. This tool has been validated and used in several studies in Vietnam [32, 33]. The questions about seven symptoms students might experience during the last two weeks, and the possible answers could be 0 - “Not at all,” 1 - “Several days,” 2 - “More than half of the day,” 3 - “Nearly every day” based on the 4-point Likert scale. The total score ranged from 0 to 21. Students with a score of 10 and above then would be defined as “Yes” - having Anxiety”; the others with lower scores would be classified as “No” - not having anxiety. The cut-off point at 10 was proved to optimize the sensitivity (89%) and specificity (82%) [31].

### Patient health questionnaire (PHQ – 9)

The 9-item Patient Health Questionnaire (PHQ-9) is a reliable and valid measure to screen for depressive disorders and evaluate depression severity [34]. The PHQ-9 was developed in 2001 by R.L. Spitzer, J.B.W. Williams, K. Kroenke, and colleagues, with support from Pfizer US Pharmaceuticals [34]. This 9-question tool was validated and utilized in many studies in Vietnam and around the world [35–37]. The questionnaire could be self-administered by the research subjects, with questions focusing on whether subjects had been bothered by any of the listed-in depressive problems. The PHQ-9 score ranged from 0 to 27 since each question could be scored 0 - “Not at all,” 1 - “Several days,” 2 - “More than half the days,” or 3 - “Nearly every day.”The cut points of 5, 10, 15, and 20 were recommended to categorize the severity of depressive symptoms: none/minimal (0–4), mild [5–9], moderate [10–14], moderately severe [15–19], and severe [20–27, 38]. Using the cut-off point of 10 – with 88% specificity and 88% sensitivity [34], students would be categorized into 2 groups “Have depression” (scoring 10 and above) and “Not having depression” (0–9).

### 12-item short-form health survey (SF-12)

The quality of life of students was determined using the 12-item SF-12 Health Survey – an abbreviated version of SF-36, which was first developed by John E. Ware, Jr, Mark Kosinski and Susan D. Keller in 1995 [39]. SF-12, comprising of 12 questions, was reported to be valid and sensitive to assessing the physical and mental health of the research subject [40]. Thanks to its shortness, SF-12 is quickly becoming a great option for monitoring the health of both general and specific populations [39]. There are several studies on Vietnamese people using SF-12 [41, 42]. As a subset of SF-36, SF-12 measures eight concepts, including physical functioning (PF), role limitations because of physical health problems (RP), bodily pain (BP), general health (GH), vitality (VT), social functioning (SF), role limitations because of emotional problems (RE), and mental health (MH). Physical health component (PCS) and mental health component (MCS) were calculated using norm-based methods from 8 subscales ranging from 0 to 100, with higher scores indicating better physical and health functioning. The relative validity estimates for the PCS-12 ranged from 0.43 to 0.93 (median = 0.67), and the relative validity estimates ranged from 0.60 to 107 (median = 0.97) in relation to the 36-item short-form scale [43]. Both the two summary scales, PCS-12 and MCS-12, are transformed to have a mean of 50 and a standard deviation of 10 relative to the general U.S. population [39, 44]. Using norm-based scoring and standardization for PCS-12 and MCS-12, the results can be meaningfully compared with the others and directly interpret with the general U.S. population. In our study, the SF-12 questionnaire online version was used [45] and translated into Vietnamese and then back-translated by two separate groups of experts. The original online English version and the back-translated version were qualified by another independent translator to ensure appropriateness. After being accepted by translators, the Vietnamese version would be modified to adapt to the Vietnamese language and culture. Acknowledging the question about the interference of physical health or emotional problems with social activities in the online English version used were 6 answers instead of 5 answers as used in other studies [46, 47], we regrouped answers 2 “Most of the time” and 3 “A good bit of the time” to be 2 “Most of the time”; other answers included: “All of the time” scored as 1, “Some of the time” scored as 3, “A little of the time” scored as 4, and “None of the time” scored as 5. We used analysis syntax developed by Ware J et al. for scoring the SF-12 questionnaire [39]. To ensure the robustness, sensitivity analyses were performed (see more at Supplemental 1).

### Academic motivation

Academic motivation was assessed using the Academic Motivation Scale (AMS-C 28) questionnaire designed by Vallerand et al. in 1992 [10] based on self-determination theory by Deci and Ryan, consisting of 28 items. The AMS-C 28 questionnaire was previously used in research by Tung Pham et al. on medical students in Vietnam and in the research on Vietnamese university students by To Lan or in the study of Quang Ngoc Nguyen and Luot Van Nguyen [48–50]. To ensure linguistic accuracy, in our research, the AMS-C 28 questionnaire was translated and back-translated by experts fluent in English and Vietnamese in our research. The scale ranged from “Does not correspond at all” to “Correspond exactly” and was scored from 1 to 7 on the Likert scale. The assessment questionnaire consisted of 28 questions subdivided into 7 subscales:

Intrinsic motivation included: (1) To know (IMK): the individuals perform activities for the pleasure and satisfaction experienced while learning, exploring, and trying to archive knowledge; (2) Toward accomplishment (IMA): the individuals perform activities for the pleasure and satisfaction experienced while attempting to accomplish or create something; (3) To experience stimulation (IMS): the individuals perform activities for the sensory pleasure, aesthetic stimulating experiences derived from the activities.

Extrinsic motivation included 3 components: 4) External regulation (EMR): the individuals perform activities for rewards and constraints; 5) Introjected (EMIN): the individuals perform activities with the internalization of past external contingencies; 6) Identified (EMID): the individuals perform activities as the activities are judged valued and essential for the individuals by themselves.

Finally, Amotivation (AM): the individuals do not perceive contingencies between outcomes and their actions.

The scale was proved to be reliable with the internal consistencies (Cronbach’s alpha values) for subscales ranging from 0.83 to 0.86 only the scale of EMID (Extrinsic motivation: Identified) having a Cronbach alpha of 0.62, by a study by Vallerand et al. in 1992 [10]. In our study, the internal consistencies (Cronbach’s alpha values) for each subscale ranged from 0.87 to 0.93, with the overall scale reliability coefficient was 0.9. We used the self-determination index to represent the collective levels of motivation among survey-participated students; this would facilitate a more straightforward interpretation and provide a deeper insight into the overall motivational climate [12, 49]. The self-determinant index (SDI) deprived of AMS-28 scale would be calculated using the formula: *[(2×Intrinsic Motivation) + (1×Identified Regulation) – (External Regulation + Introjected Regulation)/2–(2×Amotivation)]* and ranges from – 18 (little self-determined) to 18 (extreme self-determined), the higher SDI score, the more intrinsic the participant is [49, 51]. Based on SDI, students would be divided into two groups: self-determined academic motivation profile (SDI > 0 – closer to intrinsic motivation) and non-self-determined academic motivation (SDI ≤ 0 – closer to amotivation) [10, 52, 53].

### Data management and statistical analysis

Data were analyzed using STATA/MP 17.0 software. Median, interquartile range of non-normally distributed quantitative variables and frequency, percentage of qualitative variables were calculated. Non-normally distributed variables were compared using the Mann-Whitney U test, and Chi-square test was used to compare the differences between categorical groups. We consider p-value less than 0.05 as statistically significant in this study.

The graph of answers for AMS–C 28 in the paper was created using R version 4.2.3 and the Likert-plot package [54]. As the missing percentage of our research was considerable, 26,6% – which may lead to bias and loss of information [55], we imputed the missing data in STATA MP 17.0 using mi chained – where a sequence of univariate imputation models was used to impute variables, following the practical guide and flowcharts suggested by Jakobsen et al. [56].

We created a simple causal diagram (DAG – directed acyclic graph) based on the literature review to assess the association between factors. The utilization of DAG has been proven to be effective in choosing the covariates compared to the traditional statistical approaches in minimizing the magnitude of the bias in the estimate [57–59]. For two binary variables, self-reported anxiety and depression, we estimated Prevalence Ratios (PRs) using fit-modified Poisson regression models with robust error variances. These models would produce unbiased estimates for high prevalent outcomes compared to logistic regression [60–62], as models using log-binomial regression models usually failed to converge [63]. For two continuous components of the quality of life scale, we reviewed the diagnostic plot using the residual-versus-fitted plot in STATA to assess the appropriateness of the model, then ran the linear regression for the model of physical health score and mental health score [64–66].

### Ethical clearance

The study was approved by the Hanoi University of Public Health Ethics Council, registration number: 430/2018/YTCC-HD3. All participants were informed comprehensively about the survey before giving consent by clicking the “I agree to participate” button on the consent page of the questionnaire. The Institutional Review Boards (IRB) approved this consent procedure. Student participation was completely voluntary and reserved the right to discontinue or refuse to continue to participate in research at any time.

## Results

**Table 1:** Demographic factors of research participants

Columns by: Majors	Doctor	Bachelor	Total	*P*-value	Missings / *N* (Pct)
n (%)	1305 (75.7)	418 (24.3)	1723 (100)		0 / 1723 (0.0)
Demographic					
Gender, n (%)					
Male	679 (52.0)	61 (14.6)	740 (42.9)	**< 0.001** ^**a**^	
Female	626 (48.0)	357 (85.4)	983 (57.1)		0 / 1723 (0.0)
Ethnics, n (%)					
Kinh	1238 (94.9)	387 (92.6)	1625 (94.3)	0.08^a^	
Others	67 (5.1)	31 (7.4)	98 (5.7)		0 / 1723 (0.0)
Perceived financial status, n (%)					
Not having financial burden	1113 (87.8)	337 (81.8)	1450 (86.4)	**0.002** ^**a**^	
Having financial burden	154 (12.2)	75 (18.2)	229 (13.6)		44 / 1723 (2.6)
Type of housemate, n (%)					
Living alone	203 (15.7)	30 (7.3)	233 (13.7)	**< 0.001** ^**a**^	
Living with family	385 (29.8)	138 (33.4)	523 (30.7)		
Living with friends	680 (52.6)	231 (55.9)	911 (53.4)		
Others	25 (1.9)	14 (3.4)	39 (2.3)		17 / 1723 (1.0)
Marial status, n (%)					
Single	1270 (97.8)	415 (99.3)	1685 (98.1)	0.14^b^	
Married	15 (1.2)	2 (0.5)	17 (1.0)		
Others	14 (1.1)	1 (0.2)	15 (0.9)		6 / 1723 (0.3)
Academic factors					
Academic year, n (%)					
First year	702 (53.8)	304 (72.7)	1006 (58.4)	**< 0.001** ^**a**^	
Final year	603 (46.2)	114 (27.3)	717 (41.6)		0 / 1723 (0.0)
Health-related factors					
Academic motivation, n (%)					
Non-self determined	652 (64.0)	147 (50.5)	799 (61.0)	**< 0.001** ^**a**^	
Self-determined	366 (36.0)	144 (49.5)	510 (39.0)		414 / 1723 (24.0)
Self-reported generalized anxiety disorder, n (%)					
No	1020 (89.2)	332 (93.3)	1352 (90.2)	**0.026** ^**a**^	
Yes	123 (10.8)	24 (6.7)	147 (9.8)		224 / 1723 (13.0)
Self-reported depression, n (%)					
No	948 (81.9)	301 (85.0)	1249 (82.6)	0.169^a^	
Yes	210 (18.1)	53 (15.0)	263 (17.4)		211 / 1723 (12.2)
Physical health score (PCS), median (IQR)	50.27 (45.09; 54.19)	49.22 (43.95; 53.23)	50.17 (44.48; 54.03)	**0.023** ^**c**^	206 / 1723 (12.0)
Mental health score (MCS), median (IQR)	43.80 (38.53; 48.11)	43.45 (37.29; 49.43)	43.56 (38.19; 48.25)	0.896^**c**^	206 / 1723 (12.0)

**Statistical test and abbreviation**^a^ Chi-square test for categorical variable - display as n(%); ^b^ Fisher-exact test for categorical variable - display as n(%);

^c^ Mann-Whitney U test for continuous-skewed variable - display as median(IQR); The bold p-value indicated statistical significance (*p* < 0.05)

### IQR: interquartile range

Table 1 shows the socio-demographic characteristics of the study sample; overall, 75.7% of the participants were doctor students and 24.3% were bachelor students. The majority was from Kinh ethnic group (94.3%); with only 13.6% self-report having financial problems, and more than half of respondents lived with friends (53.4%). Regarding the type of housemate, most of students were living with friends with 53.4%. The gender distribution in terms of academic factors was divergent as the percentage of male students who majored in doctor was higher than in female peers (*p* < 0.001).

The prevalence of students being screened of having anxiety and depression was 9.8% and 17.4%, respectively. Regarding quality of life, the overall PCS score and MCS score were 50.17 (44.48; 54.03) and 43.56 (38.19; 48.25), respectively. Doctoral students had a higher physical health score compared to bachelor students.

**Table 2:** Academic motivation subscales by major

Academic Major	Doctor	Bachelor	Total	*p*-value
**Intrinsic Motivation, median (IQR)**				
To know (IMK)	4.00 (3.25–4.75)	4.00 (3.25–4.75)	4.00 (3.25–4.75)	0.25^a^
Towards Accomplishment (IMA)	4.00 (3.00–4.50)	4.00 (3.00–4.50)	4.00 (3.00–4.50)	0.19^a^
To experience stimulation (IMS)	4.00 (3.00–4.25)	3.50 (2.50–4.00)	4.00 (3.00–4.25)	**< 0.001** ^**a**^
**Extrinsic Motivation, median (IQR)**				
Identified (EMID)	4.00 (3.50–5.00)	4.12 (3.75–5.00)	4.00 (3.50–5.00)	0.11^a^
Introjected (EMIN)	3.75 (2.75–4.00)	3.50 (2.50–4.00)	3.75 (2.75–4.00)	**0.02** ^**a**^
External Regulation (EMR)	4.00 (3.50–4.75)	4.00 (3.25–5.00)	4.00 (3.50–4.75)	0.36^a^
**Amotivation, median (IQR)**				
Amotivation (AM)	3.25 (1.88–4.00)	2.50 (1.50–4.00)	3.00 (1.75–4.00)	**< 0.001** ^**a**^
Self-determination Index, median (IQR)				
**Self-determination index (SDI)**	0.44 (0.00–3.88)	1.71 (0.00–5.21)	0.67 (0.00–4.23)	0.002^a^

**Statistical test and abbreviation **^a^ Mann-Whitney U test for continuous-skewed variable - display as median (IQR); The bold p-value indicated statistical significance (*p* < 0.05); IQR: interquartile range

Table 2 shows the answer to academic motivation subscales by academic majors. The proportion of answers by each question in the AMS-C 28 questionnaire was described in eFigure 1 – Supplemental 1. Overall, IMK, IMA, EMID, and EMR were similar between academic disciplines. However, there were some noticeable distinctions across academic majors regarding IMS, EMIN, AM, and self-determination index (SDI). Regarding domains of AMS–C 28, the median score of bachelor students was lower than doctor students IMS (3.50 (2.50–4.00) vs. 4.00 (3.00–4.25); *p* < 0.001) and EMIN (3.75 (2.75–4.00) vs. 3.50 (2.50–4.00); *p* = 0.02). Students who majored in doctor ranked higher in AM score (3.25 (1.88–4.00) vs. 2.50 (1.50–4.00), *p* < 0.001) and lower in SDI score compared to those who majored in bachelor 0.44 (0.00–3.88) vs. 1.71 (0.00–5.21), *p* = 0.002.

**Table 3:** Distribution of academic motivation profile

Column by: Academic Motivation Profile	Non-self-determined	Self-determined	Total	*p*-value
Number of participants	496 (37.9)	813 (62.1)	1309 (100.0)	
% (95%CI)	37.9 (35.3–40.5)	62.1 (59.5–64.7)		
Demographic factors				
Gender, n (%)				
Male,	237 (41.9)	329 (58.1)	566 (100.0)	
Female	259 (34.9)	484 (65.1)	743 (100.0)	**0.01** ^**a**^
Ethnic group, n (%)				
Kinh	472 (38.0)	771 (62.0)	1243 (100.0)	
Others	24 (36.4)	42 (63.6)	66 (100.0)	0.793^a^
Perceived financial status, n (%)				
Not having financial burden	430 (37.8)	707 (62.2)	1137 (100.0)	
Having financial burden	64 (39.0)	100 (61.0)	164 (100.0)	0.766^a^
Type of housemate, n (%)				
Living alone	80 (42.6)	108 (57.4)	188 (100.0)	
Living with family	155 (38.2)	251 (61.8)	406 (100.0)	
Living with friends	251 (37.1)	426 (62.9)	677 (100.0)	
Others	8 (26.7)	22 (73.3)	30 (100.0)	0.315^a^
Marital status, n (%)				
Single	488 (37.9)	799 (62.1)	1287 (100.0)	
Married	4 (33.3)	8 (66.7)	12 (100.0)	
Others	3 (33.3)	6 (66.7)	9 (100.0)	> 0.99^b^
Academic factors				
Academic major, n (%)				
Doctor	405 (39.8)	613 (60.2)	1018 (100.0)	
Bachelor	91 (31.3)	200 (68.7)	291 (100.0)	**0.008** ^**a**^
Academic year, n (%)				
First year	232 (31.1)	515 (68.9)	747 (100.0)	
Final year	264 (47.0)	298 (53.0)	562 (100.0)	**< 0.001** ^**a**^
Health-related factors				
Self-reported generalized anxiety disorder, n (%)				
No	432 (37.1)	731 (62.9)	1163 (100.0)	0.086^a^
Yes	55 (45.1)	67 (54.9)	122 (100.0)	
Self-reported depression, n (%)				
No	375 (35.3)	687 (64.7)	1062 (100.0)	**< 0.001** ^**a**^
Yes	109 (49.8)	110 (50.2)	219 (100.0)	
Physical health score (PCS), median (IQR)	48.90 (43.52; 53.48)	50.70 (45.56; 54.30)	(44.55; 54.11)	**< 0.001** ^**c**^
Mental health score (MCS), median (IQR)	42.32 (36.96; 46.74)	44.82 (39.05; 49.38)	(38.20; 48.30)	**< 0.001** ^**c**^

**Statistical test and abbreviation **^a^ Chi-square test for categorical variable - display as n (%); ^b^ Fisher-exact test for categorical variable - display as n (%);^c^ Mann-Whitney U test for continuous-skewed variable - display as median (IQR); The bold p-value indicated statistical significance (*p* < 0.05); IQR: interquartile range

In Table 3, in a total of 1309 responses, 813 students were classified as self-determined, accounting for 62.1%. The percentage of self-determined female respondents was higher than male counterparts (65.1% vs. 58.1%; *p* < 0.001). For other demographic factors, including ethnic group, perceived financial status, type of housemate, and marital status, no significant difference was found between the two groups of academic motivation profile. Regarding academic majors, the percentage of bachelor students being classified as self-determined was higher than the group of doctor students (68.7% vs. 60.2%; *p* = 0.008). First-year students seemed to be more self-determined than those in final year (68.9% vs. 53.0%; *p* < 0.001). Concerning health-related factors, 50.2% of students self-reporting depression and 54.9% of students self-reporting anxiety coming from the “self-determined” group, and 49.8% students with depression and 45.1% with anxiety belong to the non-self-determined group (*p* < 0.001). Besides, in both physical and mental health components of quality-of-life, “self-determined” students had a higher score than “non-self-determined” ones, 50.70 (45.56; 54.30) vs. 48.90 (43.52; 53.48), *p* < 0.001 and 44.82 (39.05; 49.38) vs. 42.32 (36.96; 46.74), *p* < 0.001, respectively.

**Table 4:** Regression model on generalized anxiety disorder (GAD-7), depression (PHQ-9), and quality of life

		GAD-7		PHQ-9		PCS		MCS	
		PR	95%CI	PR	95%CI	Coef	95%CI	Coef	95%CI
**Academic motivation**	Self-determined	REF		REF		REF		REF	
	Non-self-determined	1.44	1.03–2.01^a^	1.62	1.27–2.06^a^	-1.47	-2.19 - -0.74^a^	-2.45	-3.34 - -1.55^a^

REF: reference value ^a^*p* < 0.05 All Prevalence Ratios were adjusted using Poisson multivariate regression model

Table 4 shows the associations between academic motivation profile and health-related outcomes, including anxiety screened by GAD-7, depression by PHQ-9, and quality of life screened by SF-12 (MCS) (see more from eTable 2 to eTable 7.2 – Supplemental 1). Students with non-self-determined academic motivation presented a higher probability of being screened positive for anxiety disorder (PR = 1.44, 95%CI: 1.03–2.01) compared to those counterparts being listed as self-determined. For respondents reported to be non-self-determined, the probability of being screened positive for depression was 1.62 times greater than those with a self-determined academic profile (PR = 1.62; 95%CI: 1.27–2.06). In terms of quality of life, we notice no specific pattern in the residual analysis of two linear regression models of PCS and MCS. (eFigures 2 and 3 – Supplemental 1). Academic motivation was also associated with both PCS and MCS as students were non-self-determined, physical (Coef = -1.47, 95%CI: -2.19 - -0.74) and mental health functioning (Coef = -2.45, 95%CI: -3.34 - -1.55) tended to decrease, leading to poorer quality of life.

We also presented factors associated with academic motivation profile in eTable 8 – Supplemental 1.

## Discussion

Our research showed that the percentage of non-self-determined students was 37.9 (95%CI: 35.3–40.5), higher than the result of a prior study conducted in the same school [48]. The observed difference in the results could be the sequence of the fact that our research recruited first-year and final-year students from both bachelor’s and doctor’s programs, while the previous study focused on three cohorts of medical students in the doctor’s program from the fourth to sixth year. Considering academic motivation was different across academic years and majors, we could expect differences in the prevalence of students reporting self-determined and non-self-determined academic motivation in different cohorts [67–69].

Regarding demographic and academic-related factors, our study found that academic motivation was higher in females and first-year students, which were similar to several studies [70–72]. This observation could be explained by earlier studies, that suggested females having higher levels of internal control and lower levels of external control; therefore, are more intrinsically motivated and less extrinsically motivated than males [70, 73, 74]. In terms of academic years, first-year students tended to display higher levels of self-determination compared to final year students. The finding of Brouse and colleagues, in an article published in 2010, supports our research result, as they stated that both intrinsic and extrinsic motivation declined as students progressed through college years [72]; which corresponds with opinions mentioned in other related studies that, generally, when ones advance through their academic years, their levels of motivation diminish, and they become less and less self-determined [75, 76]. The higher academic motivation observed in first-year students can be attributed to their higher consciousness about learning, along with the excitement and enthusiasm that come with the beginning of university life [75].

The finding of our research revealed distinct differences between doctor students and bachelor students; doctoral students demonstrated higher level of IMS domain and EMIN domain. However, doctor students also exhibited higher Amotivation score, which might be a considerable factor leading to an overall lower self-determination index compared to bachelor students. This disparity may come from the difference in gender distribution as while the gender ratio among doctoral students was relatively the same for both male and female counterparts, there was a significant female predominance in the number of bachelor students (eTable 8 – Supplemental 1) [48, 77–79]. Besides, in Vietnam’s medical education setting, the fact that doctor students had to take extra two years compared to those in bachelor’s programs to fulfill the training curriculum, with more exhausting training and competitive graduates attending residency programs after graduation might also contribute to this disparity [4]. This prolonged, intensive demanding curriculum could be a possible factor leading to the decline in academic motivation of medical students, resulting in less self-determined students [24, 75].

Our research found a significant association between academic motivation and other health-related outcomes, including anxiety disorder, depression, and quality of life. Non-self-determined students were more likely to have depression and anxiety disorder. These findings supported previous cross-sectional studies among Korean [18], Chinese [19], and Vietnamese medical students [48] that found negative associations between academic motivation and stress and depression. Longitudinal studies also revealed the protective role of academic motivation and personal goals against psychological distress symptoms in university students [80–83]. As the prevalence of depression among medical students is relatively high and is associated with low academic motivation, interventions aimed at raising students’ academic motivation should be carried out [84]. Regarding quality of life, non-self-determined academic motivation also had significant negative associations with both components of quality of life. This finding is similar to previous related research that showed a positive relationship between quality of life and academic motivation [20, 21, 85]. Our study also extended these findings by examining Vietnamese students specializing in medical and health sciences. To our best knowledge, no previous study has examined the association between academic motivation and quality of life in a Southeast Asia medical school context; the implementation of the Vietnamese version of SF-12 and AMS-C 28 also provided insights supporting previous and future studies.

Despite the efforts of medical schools to implement selection procedures aimed at choosing the most motivated students for their programs, the role of motivation in the selection process remains underexplored [1]. In the United States of America and the Netherlands, the medical admission procedures go beyond academic performance, with substantial time and resources devoted to evaluating students’ motivation, through a variety of tools (personal statement, interviews, or multiple interviews) [1, 86, 87]. However, in the context of Vietnamese medical education during this study, similar to China and Japan, most students choose to pursue higher education directly after graduating from high school [3, 5, 88]. Medicine was always considered a top profession choice, and parental influence towards students’ career decisions, which is inevitable, is frequently the primary drive for most students to apply for medical schools, over intrinsic motivation [4, 89, 90]. This could result in elevated levels of anxiety, depression and poorer quality of life in students later [19, 90]. Despite this, the admission process in medical institutions must still be based on each nation’s policy and socio-demographic conditions [88].

Our study found the association between academic motivation and depression, anxiety, and quality of life of medical students and students majoring in health sciences - which resembles the findings of previous research. With medicine being a major with a prolonged training curriculum, with demanding workload and intensive training schedule; the academic motivation of students was surveyed to be lowered by years of learning, as students adapt [26, 53, 72]. Still, the prevalence of depression, anxiety, and poor quality of life was relatively high in medical students [26, 91, 92]. Recently, countries started to initiate a shift in their approach by introducing some changes to the admission process. The Shanghai government in China imposed a pilot program allowing 9 universities, 11 admission units to practice holistic admission with rounds of interview. In Vietnam, with VinUniversity, interviewing is also required for the selection of students to College of Health Sciences [6, 93]. With these alterations which reflect a more thorough approach in admission process, we recommend considering the assessment of motivation in the admission process at medical schools to recruit highly motivated students for medical training programs, to promote the well-being of future healthcare professionals, and to foster their success in their academic journey.

### Limitation and strength

The strengths of our study are having a representative sample, a reasonable overall response rate of 85.1%, and using good screening tools. However, this study has certain limitations that should be considered. Firstly, the data in our research is from first and final year students from one medical school that will not be representative or accurately reflect all academic year and doctoral and bachelor of health sciences students in other countries. Secondly, as the survey was conducted after student finished their exam, which may introduce some bias due to students might experience tiredness. However, we got a higher response rate compared to online survey-method, ensuring a more comprehensive dataset. Thirdly, although the study had a large sample size, the distribution of participants by major was uneven, such that the students in the doctor branch made up a significant proportion of the sample. However, this discrepancy is unavoidable because the number of candidates selected between majors was not distributed equally according to the university’s selection criteria; future studies should target larger samples of students taking bachelor’s majors to address this issue. Fourthly, we acknowledge that the cross-sectional study design has limitations in establishing causation, and future longitudinal studies should verify and provide additional insights for potential interventions to improve medical learning efficacy and teaching quality. Besides, as the SF-12 questionnaire and AMS-C 28 questionnaire had not been validated in Vietnam at the time we conducted this survey, the tools were translated into Vietnamese and back-translated into English by separated groups of experts, and Vietnamese versions were modified to adapt to the Vietnamese language and culture. Therefore, this process could probably reduce the validity of the tools and require further validation efforts. As participants’ interpretation and understanding of the questions may impact their response, the result may be inaccurate as some students had different ways of interpreting the questions; however, we expect this measurement error to be non-differential as the actual prevalence ratio was expected to be larger than reported in our research. Additionally, due to the use of multiple imputation methods to estimate missing observations, which relied on assumptions of variable relationships, it might have resulted in biased estimates that did not reflect the data structure.

## Conclusion

This study showed that students who were females, in the first year of academic program and pursuing a Bachelor of Health Sciences degree tended to be more self-determined compared to other counterparts. Academic motivation was also found to be associated with depression, anxiety, and quality of life of doctoral and bachelor of health science students in a Vietnamese context. Students with higher levels of self-determination were inclined to experience a lower prevalence of depression, anxiety, and diminished quality of life. Further studies should verify and provide further insights into academic motivation as well as the use of academic motivation as a criterion during the selection process of medical schools to improve the quality of medical education in the future.

## Electronic supplementary material

Below is the link to the electronic supplementary material.

Supplementary Material 1

## Data Availability

According to our application to the Institutional Review Board (IRB) of Hanoi University of Public Health, the data cannot be shared publicly because of ethical restrictions to protect the confidentiality of the participants imposed by the IRB. A de-identified dataset is available for researchers who meet the criteria for access to confidential data. Requests for data should be submitted to Institutional Review Board of Hanoi University of Public Health (irb@huph.edu.vn) and the corresponding author, Dr. Thanh Tung Pham (tung.pt@vinuni.edu.vn).

## Abbreviations

AM: Amotivation
AMS-C 28: Academic Motivation Scale
DAG: Directed acyclic graph
EMID: Extrinsic Motivation – identified
EMIN: Extrinsic motivation – introjected
EMR: Extrinsic Motivation – regulation
GAD-7: Generalized Anxiety Disorder Questionnaire
HMU: Hanoi Medical University
IMA: Intrinsic motivation – towards accomplishment
IMK: Intrinsic motivation – to know
IMS: Intrinsic motivation – to experience stimulation
MCS: Mental health component
MH: Mental health
MOET: Ministry of Education and Training
PCS: Physical health component
PHQ-9: Patient Health Questionnaire
PR: Prevalence ratio
SDI: Self-determination index
SF-12: 12-item short-form Health Survey
