# Supplementary material for: Academic motivation in association with mental health and quality of life among medical and health science students: a survey in Vietnam
Source: BMC Med Educ. 2025 Jul 1;25:986. doi: 10.1186/s12909-025-07368-4 (PMC12219361; doi:10.1186/s12909-025-07368-4)
Supplement: Supplementary file 1 — Supplementary Material 1 [file 12909_2025_7368_MOESM1_ESM.docx]

**Supplemental Online Content**

[eMethods.](#_Toc196833428)

[eTable 0. Criteria for participants selection](#_Toc196833429)

[eTable 1: Demographic factors of research participants by genders](#_Toc196833430)

[eFigure 1. Distribution of Academic Motivation Questions](#_Toc196833431)

[eFigure 2. Residual plot for Physical Health Component (PCS)](#_Toc196833432)

[eFigure 3. Residual plot for Mental Health Component (MCS)](#_Toc196833433)

[eTable 2. Poisson Regression model on Generalized Anxiety Disorder (GAD-7)](#_Toc196833434)

[eTable 3. Poisson Regression model on Generalized Anxiety Disorder (GAD-7) without multiple imputation](#_Toc196833435)

[eTable 4. Poisson Regression model on Depression (PHQ-9)](#_Toc196833436)

[eTable 5. Poisson Regression model on Depression (PHQ-9) without multiple imputation](#_Toc196833437)

[eTable 6.1. Regression model on Quality of Life (SF-12)](#_Toc196833438)

[eTable 6.2 Regression model on Quality of Life (SF-12)*](#_Toc196833439)

[eTable 7.1. Regression model on Quality of Life (SF-12) without multiple imputation](#_Toc196833440)

[eTable 7.2. Regression model on Quality of Life (SF-12) without multiple imputation*](#_Toc196833441)

[eTable 8. Poisson Regression model on Academic Motivation](#_Toc196833442)

[eTable 9. Poisson Regression model on Academic Motivation without multiple imputation](#_Toc196833443)

[eTable 10. Logistic Regression model on Generalized Anxiety Disorder (GAD-7)](#_Toc196833444)

[eTable 11. Logistic Regression model on Generalized Anxiety Disorder (GAD-7) without multiple imputation](#_Toc196833445)

[eTable 12. Logistic Regression model on Depression (PHQ-9)](#_Toc196833446)

[eTable 13. Logistic Regression model on Depression (PHQ-9) without multiple imputation](#_Toc196833447)

[eTable 14. Logistic Regression model on Academic Motivation](#_Toc196833448)

[eTable 15. Logistic Regression model on Academic Motivation without multiple imputation](#_Toc196833449)

# eMethods.

**Study setting, sampling**

For the new 2018 cohort at HMU, the target was 600 students recruited in Doctor of General Medicine, 100 students in Preventive Medicine, 50 students in Traditional Medicine, 60 students in Nutrition Science, 50 students in Optometry, 50 students in Laboratory Medicine, 30 students in Public Health, and 100 students in Nursing.

The response rate of our study by majors is described in the table below:

# eTable 0. Criteria for participants selection

| **Criteria for selection** | **Majors included in the study** | **Students invited** | **Students participated** | **Response rate** |
| --- | --- | --- | --- | --- |
| First and final year students (6th year for doctor programs and 4th year for bachelor programs) would be recruited to join this study. | Doctor – 6-year programs | 1444 | 1305 | 90.4% |
|  | + Doctor of Traditional Medicine | 89 | 67 | 75.3% |
|  | + Doctor of Preventive Medicine | 146 | 47 | 32.2% |
|  | + Doctor of General Medicine | 1209 | 1191 | 98.5% |
|  | Bachelor – 4-year programs | 581 | 418 | 71.9% |
|  | + Nutrition Science | 113 | 58 | 51.3% |
|  | + Optometry | 106 | 59 | 55.7% |
|  | + Laboratory Medicine | 108 | 106 | 98.1% |
|  | + Public Health | 48 | 43 | 89.6% |
|  | + Nursing | 206 | 152 | 73.8% |
|  | **Final sample size** | 2025 | 1723 | 85.1% |

**Supplemental Tables and Figures**

# eTable 1: Demographic factors of research participants by genders

| **Columns by: Gender** | **Male** | **Female** | **Total** | **p-value** | **Missings / N (%)** |
| --- | --- | --- | --- | --- | --- |
| **Overall, n (%)** | **740 (42.9)** | **983 (57.1)** | **1723 (100.0)** |  | **0/1723 (0.0)** |
| **Demographic factors** |  |  |  |  |  |
| **Age, median (IQR)** | 20.00 (19.00 – 24.00) | 19.00 (19.00 – 24.00) | 19.00 (19.00 – 24.00) | **<0.001^a^** | 94/1723 (5.5) |
| **Ethnic group, n (%)** |  |  |  |  | 0/1723 (0.0) |
| Kinh | 711 (96.1) | 914 (93.0) | 1625 (94.3) | **0.006^b^** |  |
| Others | 29 (3.9) | 69 (7.0) | 98 (5.7) |  |  |
| **Perceived financial status, n (%)** |  |  |  |  | 44/1723 (2.6) |
| Not having financial burden | 614 (85.5) | 836 (87.0) | 1450 (86.4) | 0.38^b^ |  |
| Having financial burden | 104 (14.5) | 125 (13.0) | 229 (13.6) |  |  |
| **Type of housemate, n (%)** |  |  |  |  | 17/1723 (1.0) |
| Living alone | 144 (19.6) | 89 (9.1) | 233 (13.7) | **<0.001^b^** |  |
| Living with family | 224 (30.6) | 299 (30.7) | 523 (30.7) |  |  |
| Living with friends | 354 (48.3) | 557 (57.2) | 911 (53.4) |  |  |
| Others | 11 (1.5) | 28 (2.9) | 39 (2.3) |  |  |
| **Marital status, n (%)** |  |  |  |  | 6/1723 (0.4) |
| Single | 720 (97.7) | 965 (98.5) | 1685 (98.1) | 0.17^b^ |  |
| Married | 7 (0.9) | 10 (1.0) | 17 (1.0) |  |  |
| Others | 10 (1.4) | 5 (0.5) | 15 (0.9) |  |  |
| **Academic factors** |  |  |  |  |  |
| **Academic major, n (%)** |  |  |  |  | 0/1723 (0.0) |
| Doctor | 679 (91.8) | 626 (63.7) | 1305 (75.7) | **<0.001^b^** |  |
| Bachelor | 61 (8.2) | 357 (36.3) | 418 (24.3) |  |  |
| **Academic year, n (%)** |  |  |  |  | 0/1723 (0.0) |
| Freshman | 388 (52.4) | 618 (62.9) | 1006 (58.4) | **<0.001**^b^ |  |
| Senior year | 352 (47.6) | 365 (37.1) | 717 (41.6) |  |  |
| **Health-related factors** |  |  |  |  |  |
| **Self-reported generalized anxiety disorder, n (%)** |  |  |  |  | 224/1723 (13.0) |
| No | 560 (88.3) | 792 (91.6) | 1352 (90.2) | **0.04**^b^ |  |
| Yes | 74 (11.7) | 73 (8.4) | 147 (9.8) |  |  |
| **Self-reported depression, n (%)** |  |  |  |  | 211/1723 (12.3) |
| No | 517 (79.9) | 732 (84.6) | 1249 (82.6) | **0.02**^b^ |  |
| Yes | 130 (20.1) | 133 (15.4) | 263 (17.4) |  |  |
| **Physical health score (PCS), median (IQR)** | 48.00 (43.25 – 51.45) | 46.78 (42.35 – 50.66) | 47.25 (42.68 – 51.03) | **0.003^a^** | 459/1723 (26.6) |
| **Mental health score (MCS), median (IQR)** | 33.45 (29.83 – 37.85) | 34.68 (30.08 – 38.37) | 34.19 (29.84 – 38.13) | **0.05^a^** | 459/1723 (26.6) |
| ***Statistical test and abbreviation***  *^a^ Wilcoxon rank-sum test for continuous-skewed variable - display as median (IQR);*  *^b^ Chi-square test for categorical variable - display as n (%);*  *The bold p-value indicated statistical significance (p<0.05)*  *IQR: interquartile range* | | | | | |

# eFigure 1. Distribution of Academic Motivation Questions

**
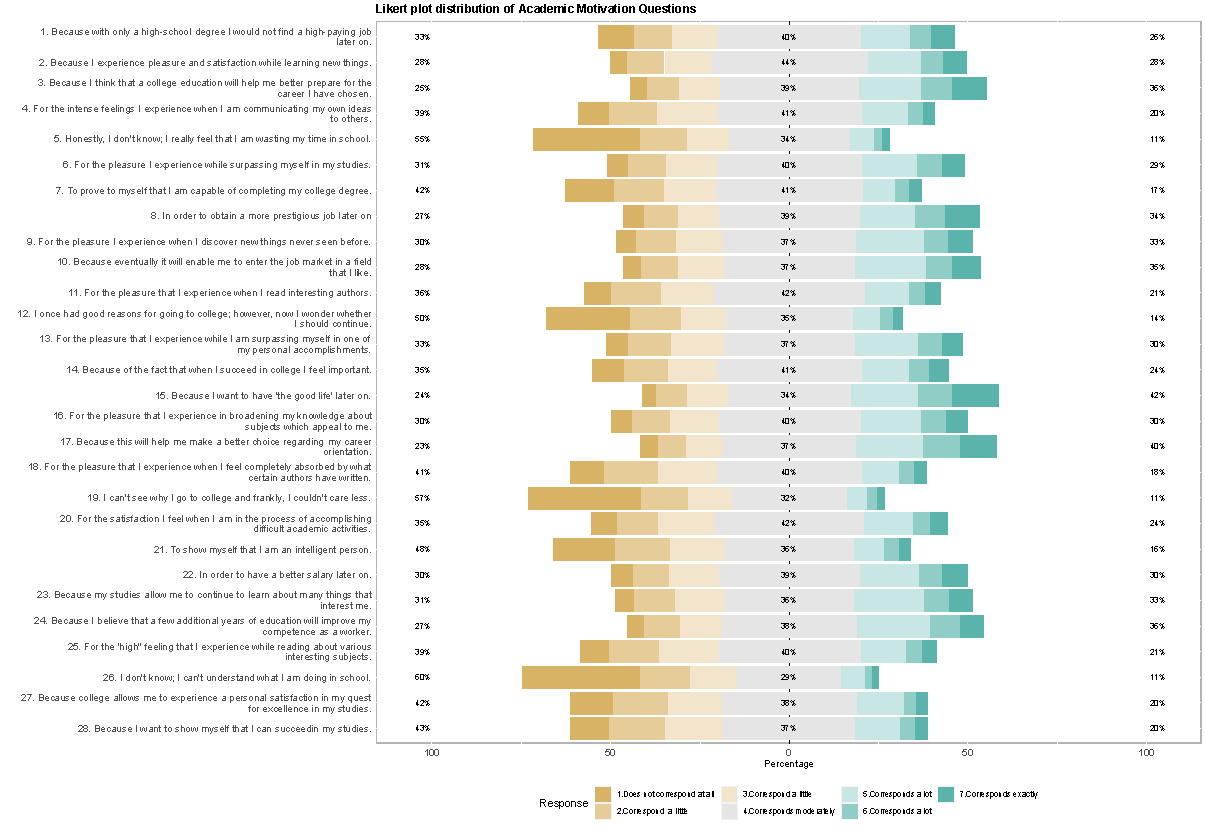
**

# eFigure 2. Residual plot for Physical Health Component (PCS)


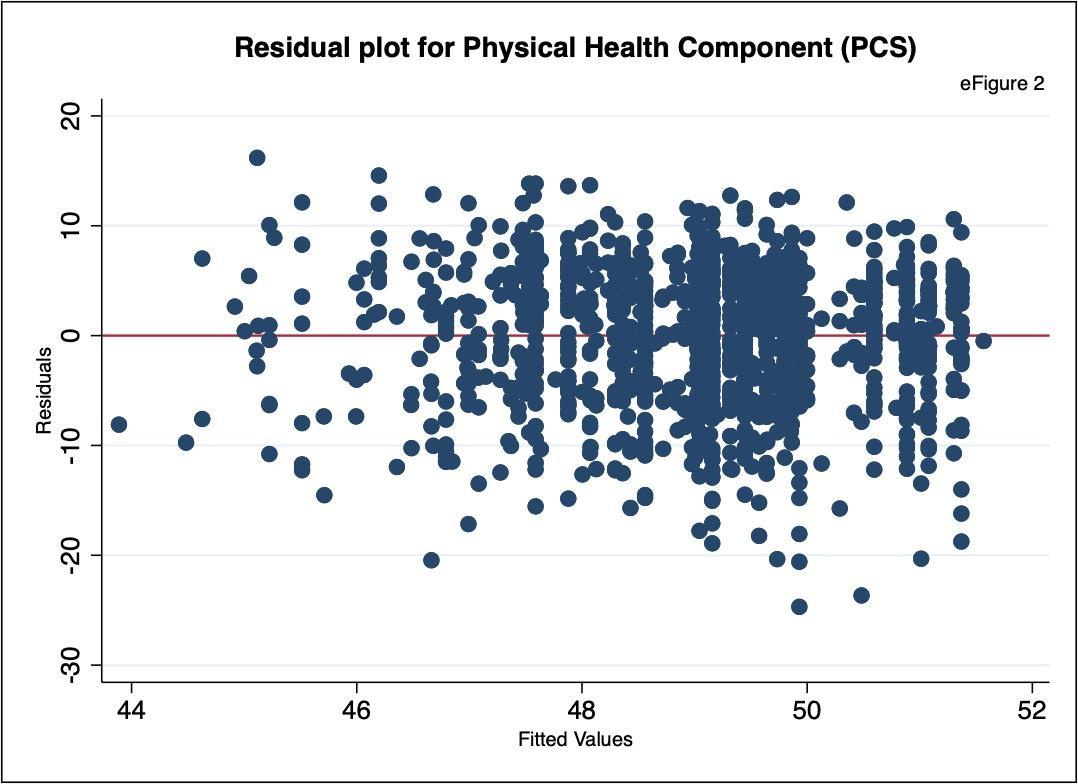


# eFigure 3. Residual plot for Mental Health Component (MCS)


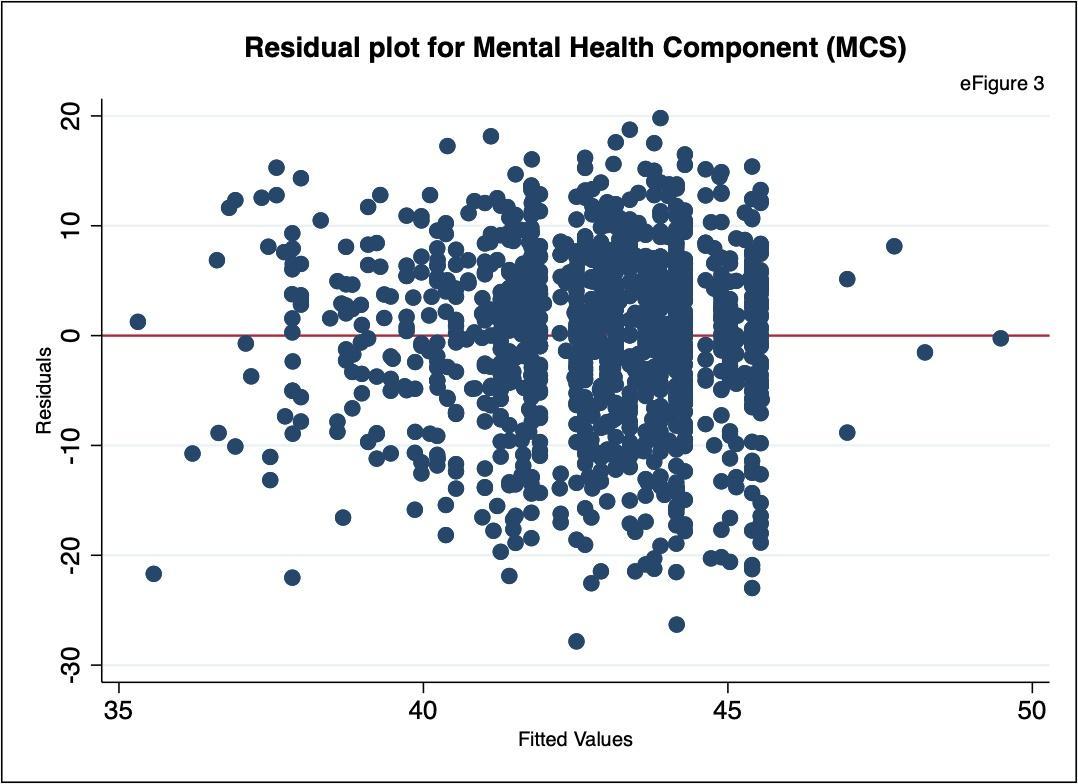


# eTable 2. Poisson Regression model on Generalized Anxiety Disorder (GAD-7)

Acknowledging the question about the interference of physical health or emotional problems with social activities in the online English version used “During the PAST 4 WEEKS, how much of the time has your physical health or emotional problems interfered with your social activities (like visiting with friends, relatives, etc.)? were 6 answers instead of 5 answers as used in other studies (42,43). We grouped answers 4 “Some of the time” and 5 “A little of the time” to be both scored as 4. Therefore, the scoring answers included: “All of the time” scored as 1, “Most of the time” or “A good bit of the time” scored as 2, “Some of the time” scored as 3, “A little of the time” scored as 4, and “None of the time” scored as 5.

|  | **GAD-7** | | |  |
| --- | --- | --- | --- | --- |
|  | **PR** | **95% CI** | **p-value** |  |
| **Academic motivation profile** |  |  |  |  |
| Self-determined | REF |  |  |  |
| Non-self-determined | 1.44 | 1.03 – 2.01 | **0.03** |  |
| **Demographic factors** |  |  |  |  |
| **Gender** |  |  |  |  |
| Male | REF |  |  |  |
| Female | 0.90 | 0.64 – 1.26 | 0.54 |  |
| **Ethnics** |  |  |  |  |
| Kinh | REF |  |  |  |
| Others | 0.57 | 0.24 – 1.39 | 0.22 |  |
| **Type of housemate** |  |  |  |  |
| Living alone | REF |  |  |  |
| Living with family | 0.97 | 0.59 – 1.60 | 0.90 |  |
| Living with friends | 0.99 | 0.62 – 1.57 | 0.95 |  |
| Others | 1.12 | 0.39 – 3.23 | 0.83 |  |
| **Marital status** |  |  |  |  |
| Single | REF |  |  |  |
| Married | 1.98 | 0.72 – 5.46 | 0.19 |  |
| Others | 1.65 | 0.43 – 6.34 | 0.46 |  |
| **Perceived financial burden** |  |  |  |  |
| Not having financial burden | REF |  |  |  |
| Having financial burden | 2.59 | 1.82 – 3.68 | **<0.001** |  |
| **Academic factors** |  |  |  |  |
| **Academic year** |  |  |  |  |
| First year | REF |  |  |  |
| Final year | 1.02 | 0.73 – 1.43 | 0.89 |  |
| **Academic major** |  |  |  |  |
| Doctor | REF |  |  |  |
| Bachelor | 0.66 | 0.42 – 1.03 | 0.07 |  |
| **Observations** | 1723 |  |  |  |
| *REF: Reference value*  *The bold p-value indicated statistical significance.* | | | | |

# eTable 3. Poisson Regression model on Generalized Anxiety Disorder (GAD-7) without multiple imputation

|  | **GAD-7** | | |  |
| --- | --- | --- | --- | --- |
|  | **PR** | **95% CI** | **p-value** |  |
| **Academic motivation profile** |  |  |  |  |
| Self-determined | REF |  |  |  |
| Non-self-determined | 1.3 | 0.90 – 1.87 | 0.16 |  |
| **Demographic factors** |  |  |  |  |
| **Gender** |  |  |  |  |
| Male | REF |  |  |  |
| Female | 0.67 | 0.46 – 0.99 | **0.04** |  |
| **Ethnics** |  |  |  |  |
| Kinh | REF |  |  |  |
| Others | 0.18 | 0.02 – 1.27 | 0.08 |  |
| **Type of housemate** |  |  |  |  |
| Living alone | REF |  |  |  |
| Living with family | 1.04 | 0.58 – 1.87 | 0.90 |  |
| Living with friends | 1.17 | 0.68 – 2.01 | 0.57 |  |
| Others | 1.03 | 0.29 – 3.65 | 0.96 |  |
| **Marital status** |  |  |  |  |
| Single | REF |  |  |  |
| Married | 3.66 | 1.31 – 10.20 | **0.01** |  |
| Others | 1.93 | 0.46 – 8.10 | 0.37 |  |
| **Perceived financial burden** |  |  |  |  |
| Not having financial burden | REF |  |  |  |
| Having financial burden | 2.41 | 1.60 – 3.64 | **<0.001** |  |
| **Academic factors** |  |  |  |  |
| **Academic year** |  |  |  |  |
| First year | REF |  |  |  |
| Final year | 1.01 | 0.70 – 1.46 | 0.94 |  |
| **Academic major** |  |  |  |  |
| Doctor | REF |  |  |  |
| Bachelor | 0.74 | 0.43 – 1.25 | 0.26 |  |
| **Observations** | 1270 |  |  |  |
| *REF: Reference value*  *The bold p-value indicated statistical significance.* | | | | |

# eTable 4. Poisson Regression model on Depression (PHQ-9)

Acknowledging the question about the interference of physical health or emotional problems with social activities in the online English version used “During the PAST 4 WEEKS, how much of the time has your physical health or emotional problems interfered with your social activities (like visiting with friends, relatives, etc.)? were 6 answers instead of 5 answers as used in other studies (42,43). We grouped answers 4 “Some of the time” and 5 “A little of the time” to be both scored as 4. Therefore, the scoring answers included: “All of the time” scored as 1, “Most of the time” or “A good bit of the time” scored as 2, “Some of the time” scored as 3, “A little of the time” scored as 4, and “None of the time” scored as 5.

|  | **PHQ-9** | | |  |
| --- | --- | --- | --- | --- |
|  | **PR** | **95% CI** | **p-value** |  |
| **Academic motivation profile** |  |  |  |  |
| Self-determined | REF |  |  |  |
| Non-self-determined | 1.62 | 1.27 – 2.06 | **<0.001** |  |
| **Demographic factors** |  |  |  |  |
| **Gender** |  |  |  |  |
| Male | REF |  |  |  |
| Female | 0.86 | 0.67 – 1.11 | 0.26 |  |
| **Ethnics** |  |  |  |  |
| Kinh | REF |  |  |  |
| Others | 0.97 | 0.59 – 1.62 | 0.92 |  |
| **Type of housemate** |  |  |  |  |
| Living alone | REF |  |  |  |
| Living with family | 0.87 | 0.59 – 1.28 | 0.48 |  |
| Living with friends | 0.97 | 0.69 – 1.37 | 0.86 |  |
| Others | 1.06 | 0.47 – 2.39 | 0.88 |  |
| **Marital status** |  |  |  |  |
| Single | REF |  |  |  |
| Married | 1.18 | 0.41 – 3.42 | 0.76 |  |
| Others | 1.64 | 0.63 – 4.30 | 0.31 |  |
| **Perceived financial burden** |  |  |  |  |
| Not having financial burden | REF |  |  |  |
| Having financial burden | 2.07 | 1.57 – 2.73 | **<0.001** |  |
| **Academic factors** |  |  |  |  |
| **Academic year** |  |  |  |  |
| First year | REF |  |  |  |
| Final year | 0.95 | 0.74 – 1.21 | 0.68 |  |
| **Academic major** |  |  |  |  |
| Doctor | REF |  |  |  |
| Bachelor | 0.88 | 0.64 – 1.20 | 0.41 |  |
| **Observations** | 1723 |  |  |  |
| *REF: Reference value*  *The bold p-value indicated statistical significance.* | | | | |

# eTable 5. Poisson Regression model on Depression (PHQ-9) without multiple imputation

|  | **PHQ-9** | | |  |
| --- | --- | --- | --- | --- |
|  | **PR** | **95% CI** | **p-value** |  |
| **Academic motivation profile** |  |  |  |  |
| Self-determined | REF |  |  |  |
| Non-self-determined | 1.60 | 1.22 – 2.10 | **<0.001** |  |
| **Demographic factors** |  |  |  |  |
| **Gender** |  |  |  |  |
| Male | REF |  |  |  |
| Female | 0.75 | 0.56 – 0.99 | **0.05** |  |
| **Ethnics** |  |  |  |  |
| Kinh | REF |  |  |  |
| Others | 1.02 | 0.55 – 1.89 | 0.94 |  |
| **Type of housemate** |  |  |  |  |
| Living alone | REF |  |  |  |
| Living with family | 0.81 | 0.54 - 1.24 | 0.34 |  |
| Living with friends | 0.93 | 0.63 - 1.36 | 0.71 |  |
| Others | 1.04 | 0.43 - 2.51 | 0.93 |  |
| **Marital status** |  |  |  |  |
| Single | REF |  |  |  |
| Married | 1.54 | 0.38 – 6.33 | 0.55 |  |
| Others | 2.23 | 0.81 – 6.13 | 0.12 |  |
| **Perceived financial burden** |  |  |  |  |
| Not having financial burden | REF |  |  |  |
| Having financial burden | 2.00 | 1.45 – 2.77 | **<0.001** |  |
| **Academic factors** |  |  |  |  |
| **Academic year** |  |  |  |  |
| First year | REF |  |  |  |
| Final year | 0.97 | 0.74 – 1.28 | 0.83 |  |
| **Academic major** |  |  |  |  |
| Doctor | REF |  |  |  |
| Bachelor | 0.91 | 0.63 – 1.32 | 0.63 |  |
| **Observations** | 1270 |  |  |  |
| *REF: Reference value*  *The bold p-value indicated statistical significance.* | | | | |

# eTable 6.1. Regression model on Quality of Life (SF-12)

Acknowledging the question about the interference of physical health or emotional problems with social activities in the online English version used “During the PAST 4 WEEKS, how much of the time has your physical health or emotional problems interfered with your social activities (like visiting with friends, relatives, etc.)? were 6 answers instead of 5 answers as used in other studies (42,43). We grouped answers 4 “Some of the time” and 5 “A little of the time” to be both scored as 4. Therefore, the scoring answers included: “All of the time” scored as 1, “Most of the time” or “A good bit of the time” scored as 2, “Some of the time” scored as 3, “A little of the time” scored as 4, and “None of the time” scored as 5.

|  | **PCS** | | | **MCS** | | |
| --- | --- | --- | --- | --- | --- | --- |
|  | **Coef.** | **95% CI** | **p-value** | **Coef.** | **95% CI** | **p-value** |
| **Academic motivation profile** |  |  |  |  |  |  |
| Self-determined | REF |  |  | REF |  |  |
| Non-self-determined | -1.47 | -2.19 – -0.74 | **<0.001** | -2.45 | -3.34 – -1.55 | **<0.001** |
| **Demographic factors** |  |  |  |  |  |  |
| **Gender** |  |  |  |  |  |  |
| Male | REF |  |  | REF |  |  |
| Female | -0.48 | -1.17 – 0.21 | 0.17 | -0.19 | -1.05 – 0.68 | 0.67 |
| **Ethnics** |  |  |  |  |  |  |
| Kinh | REF |  |  | REF |  |  |
| Others | 0.67 | -0.72 – 2.05 | 0.34 | -0.95 | -2.68 – 0.78 | 0.28 |
| **Type of housemate** |  |  |  |  |  |  |
| Living alone | REF |  |  | REF |  |  |
| Living with family | 0.17 | -0.91 – 1.26 | 0.76 | 0.76 | -0.57 – 2.1 | 0.26 |
| Living with friends | -0.33 | -1.35 – 0.69 | 0.53 | 1.09 | 0.17 – 2.32 | 0.09 |
| Others | -0.86 | -3.10 – 1.39 | 0.45 | -1.28 | -4.16 – 1.6 | 0.38 |
| **Marital status** |  |  |  |  |  |  |
| Single | REF |  |  | REF |  |  |
| Married | 0.65 | -2.76 – 4.05 | 0.71 | -0.001 | -4.19 – 4.19 | 1.00 |
| Others | -1.58 | -5.28 – 2.12 | 0.40 | 4.21 | 0.5 – 9.92 | 0.08 |
| **Perceived financial burden** |  |  |  |  |  |  |
| Not having financial burden | REF |  |  | REF |  |  |
| Having financial burden | -2.12 | -3.06 – -1.19 | **<0.001** | -3.58 | -4.78 – -2.37 | **<0.001** |
| **Academic factors** |  |  |  |  |  |  |
| **Academic year** |  |  |  |  |  |  |
| First year | REF |  |  | REF |  |  |
| Final year | 1.42 | 0.75 – 2.09 | **<0.001** | 1.18 | 0.34 – 2.00 | **0.006** |
| **Academic major** |  |  |  |  |  |  |
| Doctor | REF |  |  | REF |  |  |
| Bachelor | -0.38 | -1.19 – 0.42 | 0.35 | 0.45 | -0.54 – 1.44 | 0.38 |
| **Observations** | 1723 |  |  | 1723 |  |  |
| *REF: Reference value*  *The bold p-value indicated statistical significance.* | | | | | | |

# eTable 6.2 Regression model on Quality of Life (SF-12)*

*Acknowledging the question about the interference of physical health or emotional problems with social activities in the online English version used “During the PAST 4 WEEKS, how much of the time has your physical health or emotional problems interfered with your social activities (like visiting with friends, relatives, etc.)? were 6 answers instead of 5 answers as used in other studies (42,43). We grouped answers 4 “Some of the time” and 5 “A little of the time” to be both scored as 4. Therefore, the scoring answers will be: answers “All of the time” scored as 1, “Most of the time” scored as 2, “A good bit of the time” scored as 3, “Some of the time” or “A little of the time” scored as 4, and “None of the time” scored as 5.

|  | **PCS** | | | **MCS** | | |
| --- | --- | --- | --- | --- | --- | --- |
|  | **Coef.** | **95% CI** | **p-value** | **Coef.** | **95% CI** | **p-value** |
| **Academic motivation profile** |  |  |  |  |  |  |
| Self-determined | REF |  |  | REF |  |  |
| Non-self-determined | -1.41 | -2.13 – -0.70 | **<0.001** | -2.08 | -2.95 – -1.21 | **<0.001** |
| **Demographic factors** |  |  |  |  |  |  |
| **Gender** |  |  |  |  |  |  |
| Male | REF |  |  | REF |  |  |
| Female | -0.50 | -1.18 – 0.19 | 0.15 | -0.19 | -1.02 – 0.64 | 0.66 |
| **Ethnics** |  |  |  |  |  |  |
| Kinh | REF |  |  | REF |  |  |
| Others | 0.69 | -0.68 – 2.06 | 0.33 | -0.87 | -2.53 – 0.80 | 0.31 |
| **Type of housemate** |  |  |  |  |  |  |
| Living alone | REF |  |  | REF |  |  |
| Living with family | 0.17 | -0.91 – 1.25 | 0.76 | 0.72 | -0.57 – 2.02 | 0.27 |
| Living with friends | -0.32 | -1.34 – 0.69 | 0.53 | 1.17 | -0.03 – 2.37 | 0.06 |
| Others | -0.84 | -3.06 – 1.38 | 0.46 | -1.15 | -3.94 – 1.65 | 0.42 |
| **Marital status** |  |  |  |  |  |  |
| Single | REF |  |  | REF |  |  |
| Married | 0.82 | -2.56 – 4.21 | 0.63 | 0.51 | -3.54 – 4.56 | 0.80 |
| Others | -1.59 | -5.26 – 2.07 | 0.39 | 4.51 | -0.02 – 9.05 | 0.05 |
| **Perceived financial burden** |  |  |  |  |  |  |
| Not having financial burden | REF |  |  | REF |  |  |
| Having financial burden | -2.11 | -3.04 - -1.18 | **<0.001** | -3.60 | -4.76 – -2.44 | **<0.001** |
| **Academic factors** |  |  |  |  |  |  |
| **Academic year** |  |  |  |  |  |  |
| First year | REF |  |  | REF |  |  |
| Final year | 1.42 | 0.76 – 2.08 | **<0.001** | 1.24 | 0.43 – 2.04 | **0.003** |
| **Academic major** |  |  |  |  |  |  |
| Doctor | REF |  |  | REF |  |  |
| Bachelor | -0.44 | -1.24 – 0.36 | 0.28 | 0.16 | -0.80 – 1.11 | 0.75 |
| **Observations** | 1723 |  |  | 1723 |  |  |
| *REF: Reference value*  *The bold p-value indicated statistical significance.* | | | | | | |

# eTable 7.1. Regression model on Quality of Life (SF-12) without multiple imputation

Acknowledging the question about the interference of physical health or emotional problems with social activities in the online English version used “During the PAST 4 WEEKS, how much of the time has your physical health or emotional problems interfered with your social activities (like visiting with friends, relatives, etc.)? were 6 answers instead of 5 answers as used in other studies (42,43). We grouped answers 4 “Some of the time” and 5 “A little of the time” to be both scored as 4. Therefore, the scoring answers included: “All of the time” scored as 1, “Most of the time” or “A good bit of the time” scored as 2, “Some of the time” scored as 3, “A little of the time” scored as 4, and “None of the time” scored as 5.

|  | **PCS** | | | **MCS** | | |
| --- | --- | --- | --- | --- | --- | --- |
|  | **Coef.** | **95% CI** | **p-value** | **Coef.** | **95% CI** | **p-value** |
| **Academic motivation profile** |  |  |  |  |  |  |
| Self-determined | REF |  |  | REF |  |  |
| Non-self-determined | -1.57 | -2.3 – -0.84 | **<0.001** | -2.38 | -3.29 – -1.47 | **<0.001** |
| **Demographic factors** |  |  |  |  |  |  |
| **Gender** |  |  |  |  |  |  |
| Male | REF |  |  | REF |  |  |
| Female | -0.29 | -1.04 – 0.46 | 0.45 | -0.14 | -1.08 – 0.8 | 0.77 |
| **Ethnics** |  |  |  |  |  |  |
| Kinh | REF |  |  | REF |  |  |
| Others | 0.49 | -1.11 – 2.08 | 0.55 | -0.68 | -2.67 – 1.32 | 0.51 |
| **Type of housemate** |  |  |  |  |  |  |
| Living alone | REF |  |  | REF |  |  |
| Living with family | 0.07 | -1.05 – 1.18 | 0.91 | 0.88 | -0.52 – 2.27 | 0.22 |
| Living with friends | -0.42 | -1.47 – 0.63 | 0.44 | 1.38 | -0.07 – 2.69 | **0.04** |
| Others | -1.45 | -3.89 – 1.0 | 0.25 | -1.04 | -4.1 – 2.02 | 0.51 |
| **Marital status** |  |  |  |  |  |  |
| Single | REF |  |  | REF |  |  |
| Married | -0.22 | -4.14 – 3.70 | 0.91 | -1.21 | -6.11 – 3.69 | 0.63 |
| Others | -2.37 | -6.49 – 1.75 | 0.26 | -3.94 | -1.21 – 9.1 | 0.13 |
| **Perceived financial burden** |  |  |  |  |  |  |
| Not having financial burden | REF |  |  | REF |  |  |
| Having financial burden | -2.36 | -3.43 – -1.29 | **<0.001** | -3.93 | -5.25 – -2.6 | **<0.001** |
| **Academic factors** |  |  |  |  |  |  |
| **Academic year** |  |  |  |  |  |  |
| First year | REF |  |  | REF |  |  |
| Final year | 1.44 | 0.71 – 2.16 | **<0.001** | 1.24 | 0.34 – 2.15 | **0.007** |
| **Academic major** |  |  |  |  |  |  |
| Doctor | REF |  |  | REF |  |  |
| Bachelor | -0.60 | -1.5 – 0.31 | 0.2 | -0.26 | -1.39 – 0.87 | 0.65 |
| **Observations** | 1242 |  |  | 1242 |  |  |
| *REF: Reference value*  *The bold p-value indicated statistical significance.* | | | | | | |

# eTable 7.2. Regression model on Quality of Life (SF-12) without multiple imputation*

Acknowledging the question about the interference of physical health or emotional problems with social activities in the online English version used “During the PAST 4 WEEKS, how much of the time has your physical health or emotional problems interfered with your social activities (like visiting with friends, relatives, etc.)? were 6 answers instead of 5 answers as used in other studies (42,43). We grouped answers 4 “Some of the time” and 5 “A little of the time” to be both scored as 4. Therefore, the scoring answers will be: answers “All of the time” scored as 1, “Most of the time” scored as 2, “A good bit of the time” scored as 3, “Some of the time” or “A little of the time” scored as 4, and “None of the time” scored as 5.

|  | **PCS** | | | **MCS** | | |
| --- | --- | --- | --- | --- | --- | --- |
|  | **Coef.** | **95% CI** | **p-value** | **Coef.** | **95% CI** | **p-value** |
| **Academic motivation profile** |  |  |  |  |  |  |
| Self-determined | REF |  |  | REF |  |  |
| Non-self-determined | -1.51 | -2.23 – -0.79 | **<0.001** | -2.04 | -2.92 – -1.16 | **<0.001** |
| **Demographic factors** |  |  |  |  |  |  |
| **Gender** |  |  |  |  |  |  |
| Male | REF |  |  | REF |  |  |
| Female | -0.31 | -1.06 – 0.43 | 0.41 | -0.18 | -1.09 – 0.73 | 0.69 |
| **Ethnics** |  |  |  |  |  |  |
| Kinh | REF |  |  | REF |  |  |
| Others | 0.51 | -1.07 – 2.10 | 0.52 | -0.54 | -2.47 – 1.39 | 0.58 |
| **Type of housemate** |  |  |  |  |  |  |
| Living alone | REF |  |  | REF |  |  |
| Living with family | 0.07 | -1.04 – 1.18 | 0.90 | 0.87 | -0.47 – 2.22 | 0.20 |
| Living with friends | -0.40 | -1.44 – 0.64 | 0.45 | 1.49 | 0.23 – 2.76 | **0.02** |
| Others | -1.43 | -3.85 – 0.99 | 0.25 | -0.87 | -3.82 – 2.08 | 0.56 |
| **Marital status** |  |  |  |  |  |  |
| Single | REF |  |  | REF |  |  |
| Married | 0.03 | -3.85 – 3.91 | 0.99 | 0.39 | -5.11 – 4.34 | 0.87 |
| Others | -2.35 | -6.43 – 1.73 | 0.26 | 4.37 | -0.60 – 9.34 | 0.09 |
| **Perceived financial burden** |  |  |  |  |  |  |
| Not having financial burden | REF |  |  | REF |  |  |
| Having financial burden | -2.35 | -3.40 – -1.30 | **<0.001** | -3.90 | -5.18 – -2.62 | **<0.001** |
| **Academic factors** |  |  |  |  |  |  |
| **Academic year** |  |  |  |  |  |  |
| First year | REF |  |  | REF |  |  |
| Final year | 1.43 | 0.71 – 2.15 | **<0.001** | 1.28 | 0.41 – 2.16 | **0.004** |
| **Academic major** |  |  |  |  |  |  |
| Doctor | REF |  |  | REF |  |  |
| Bachelor | -0.64 | -1.54 – 0.25 | 0.16 | -0.54 | -1.63 – 0.55 | 0.33 |
| **Observations** | 1242 |  |  | 1242 |  |  |
| *REF: Reference value*  *The bold p-value indicated statistical significance.* | | | | | | |

# eTable 8. Poisson Regression model on Academic Motivation

|  | **Self-determination index (SDI)** | | |  |
| --- | --- | --- | --- | --- |
|  | **PR** | **95% CI** | **p-value** |  |
| **Demographic factors** |  |  |  |  |
| **Gender** |  |  |  |  |
| Male | REF |  |  |  |
| Female | 0.90 | 0.75 – 1.07 | 0.23 |  |
| **Ethnic groups** |  |  |  |  |
| Kinh | REF |  |  |  |
| Others | 1.00 | 0.67 – 1.5 | 1.00 |  |
| **Type of housemate** |  |  |  |  |
| Living alone | REF |  |  |  |
| Living with family | 0.94 | 0.72 – 1.23 | 0.67 |  |
| Living with friends | 0.92 | 0.72 – 1.17 | 0.49 |  |
| Others | 0.76 | 0.37 – 1.55 | 0.45 |  |
| **Marital status** |  |  |  |  |
| Single | REF |  |  |  |
| Married | 0.78 | 0.29 – 2.11 | 0.62 |  |
| Others | 0.85 | 0.31 – 2.34 | 0.75 |  |
| **Perceived financial burden** |  |  |  |  |
| Not having financial burden | REF |  |  |  |
| Having financial burden | 1.01 | 0.79 – 1.29 | 0.93 |  |
| **Academic factors** |  |  |  |  |
| **Academic year** |  |  |  |  |
| First year | REF |  |  |  |
| Final year | 1.54 | 1.29 – 1.83 | **<0.001** |  |
| **Academic major** |  |  |  |  |
| Doctor | REF |  |  |  |
| Bachelor | 0.83 | 0.66 – 1.05 | 0.11 |  |
| **Observations** | 1723 |  |  |  |
| *REF: Reference value*  *The bold p-value indicated statistical significance.* | | | | |

#

# eTable 9. Poisson Regression model on Academic Motivation without multiple imputation

|  | **Self-determination index (SDI)** | | |  |
| --- | --- | --- | --- | --- |
|  | **PR** | **95% CI** | **p-value** |  |
| **Demographic factors** |  |  |  |  |
| **Gender** |  |  |  |  |
| Male | REF |  |  |  |
| Female | 0.91 | 0.75 – 1.10 | 0.31 |  |
| **Ethnic groups** |  |  |  |  |
| Kinh | REF |  |  |  |
| Others | 1.10 | 0.73 – 1.66 | 0.66 |  |
| **Type of housemate** |  |  |  |  |
| Living alone | REF |  |  |  |
| Living with family | 0.96 | 0.73 – 1.26 | 0.77 |  |
| Living with friends | 0.93 | 0.72 – 1.20 | 0.56 |  |
| Others | 0.75 | 0.36 – 1.56 | 0.44 |  |
| **Marital status** |  |  |  |  |
| Single | REF |  |  |  |
| Married | 0.71 | 0.23 – 2.23 | 0.56 |  |
| Others | 0.80 | 0.26 – 2.50 | 0.70 |  |
| **Perceived financial burden** |  |  |  |  |
| Not having financial burden | REF |  |  |  |
| Having financial burden | 1.05 | 0.80 – 1.37 | 0.72 |  |
| **Academic factors** |  |  |  |  |
| **Academic year** |  |  |  |  |
| First year | REF |  |  |  |
| Final year | 1.47 | 1.23 – 1.77 | **<0.001** |  |
| **Academic major** |  |  |  |  |
| Doctor | REF |  |  |  |
| Bachelor | 0.86 | 0.67 – 1.10 | 0.23 |  |
| **Observations** | 1293 |  |  |  |
| *REF: Reference value*  *The bold p-value indicated statistical significance.* | | | | |

#

# eTable 10. Logistic Regression model on Generalized Anxiety Disorder (GAD-7)

|  | **GAD-7** | | |  |
| --- | --- | --- | --- | --- |
|  | **OR** | **95% CI** | **p-value** |  |
| **Academic motivation profile** |  |  |  |  |
| Self-determined | REF |  |  |  |
| Non-self-determined | 1.52 | 1.06 – 2.19 | **0.02** |  |
| **Demographic factors** |  |  |  |  |
| **Gender** |  |  |  |  |
| Male | REF |  |  |  |
| Female | 0.89 | 0.62 – 1.27 | 0.51 |  |
| **Ethnics** |  |  |  |  |
| Kinh | REF |  |  |  |
| Others | 0.54 | 0.21 – 1.36 | 0.19 |  |
| **Type of housemate** |  |  |  |  |
| Living alone | REF |  |  |  |
| Living with family | 0.97 | 0.56 – 1.66 | 0.90 |  |
| Living with friends | 0.98 | 0.60 – 1.63 | 0.95 |  |
| Others | 1.17 | 0.36 – 3.79 | 0.79 |  |
| **Marital status** |  |  |  |  |
| Single | REF |  |  |  |
| Married | 2.46 | 0.72 – 8.43 | 0.15 |  |
| Others | 1.90 | 0.38 – 9.43 | 0.43 |  |
| **Perceived financial burden** |  |  |  |  |
| Not having financial burden | REF |  |  |  |
| Having financial burden | 3.08 | 2.07 – 4.59 | **<0.001** |  |
| **Academic factors** |  |  |  |  |
| **Academic year** |  |  |  |  |
| First year | REF |  |  |  |
| Final year | 1.03 | 0.71 – 1.48 | 0.89 |  |
| **Academic major** |  |  |  |  |
| Doctor | REF |  |  |  |
| Bachelor | 0.62 | 0.39 – 1.00 | 0.05 |  |
| **Observations** | 1723 |  |  |  |
| *REF: Reference value*  *The bold p-value indicated statistical significance.* | | | | |

#

# eTable 11. Logistic Regression model on Generalized Anxiety Disorder (GAD-7) without multiple imputation

|  | **GAD-7** | | |  |
| --- | --- | --- | --- | --- |
|  | **OR** | **95% CI** | **p-value** |  |
| **Academic motivation profile** |  |  |  |  |
| Self-determined | REF |  |  |  |
| Non-self-determined | 1.35 | 0.91 – 1.99 | 0.14 |  |
| **Demographic factors** |  |  |  |  |
| **Gender** |  |  |  |  |
| Male | REF |  |  |  |
| Female | 0.64 | 0.42 – 0.96 | **0.03** |  |
| **Ethnics** |  |  |  |  |
| Kinh | REF |  |  |  |
| Others | 0.16 | 0.02 – 1.16 | 0.07 |  |
| **Type of housemate** |  |  |  |  |
| Living alone | REF |  |  |  |
| Living with family | 1.05 | 0.56 – 1.96 | 0.88 |  |
| Living with friends | 1.20 | 0.67 – 2.14 | 0.54 |  |
| Others | 1.07 | 0.26 – 4.36 | 0.92 |  |
| **Marital status** |  |  |  |  |
| Single | REF |  |  |  |
| Married | 5.92 | 1.53 – 22.83 | **0.01** |  |
| Others | 2.28 | 0.43 – 12.20 | 0.34 |  |
| **Perceived financial burden** |  |  |  |  |
| Not having financial burden | REF |  |  |  |
| Having financial burden | 2.85 | 1.80 – 4.50 | **<0.001** |  |
| **Academic factors** |  |  |  |  |
| **Academic year** |  |  |  |  |
| First year | REF |  |  |  |
| Final year | 1.00 | 0.68 – 1.49 | 0.98 |  |
| **Academic major** |  |  |  |  |
| Doctor | REF |  |  |  |
| Bachelor | 0.71 | 0.41 – 1.24 | 0.23 |  |
| **Observations** | 1270 |  |  |  |
| *REF: Reference value*  *The bold p-value indicated statistical significance.* | | | | |

#

# eTable 12. Logistic Regression model on Depression (PHQ-9)

|  | **PHQ-9** | | |  |
| --- | --- | --- | --- | --- |
|  | **OR** | **95% CI** | **p-value** |  |
| **Academic motivation profile** |  |  |  |  |
| Self-determined | REF |  |  |  |
| Non-self-determined | 1.85 | 1.40 – 2.43 | **<0.001** |  |
| **Demographic factors** |  |  |  |  |
| **Gender** |  |  |  |  |
| Male | REF |  |  |  |
| Female | 0.83 | 0.562 – 1.11 | 0.20 |  |
| **Ethnics** |  |  |  |  |
| Kinh | REF |  |  |  |
| Others | 0.97 | 0.54 – 1.72 | 0.90 |  |
| **Type of housemate** |  |  |  |  |
| Living alone | REF |  |  |  |
| Living with family | 0.84 | 0.54 – 1.31 | 0.44 |  |
| Living with friends | 0.96 | 0.65 – 1.43 | 0.84 |  |
| Others | 1.11 | 0.43 – 2.86 | 0.84 |  |
| **Marital status** |  |  |  |  |
| Single | REF |  |  |  |
| Married | 1.28 | 0.35 – 4.70 | 0.71 |  |
| Others | 2.01 | 0.57 – 7.04 | 0.27 |  |
| **Perceived financial burden** |  |  |  |  |
| Not having financial burden | REF |  |  |  |
| Having financial burden | 2.65 | 1.89 – 3.72 | **<0.001** |  |
| **Academic factors** |  |  |  |  |
| **Academic year** |  |  |  |  |
| First year | REF |  |  |  |
| Final year | 0.94 | 0.71 – 1.24 | 0.64 |  |
| **Academic major** |  |  |  |  |
| Doctor | REF |  |  |  |
| Bachelor | 0.85 | 0.60 – 1.21 | 0.36 |  |
| **Observations** | 1723 |  |  |  |
| *REF: Reference value*  *The bold p-value indicated statistical significance.* | | | | |

#

# eTable 13. Logistic Regression model on Depression (PHQ-9) without multiple imputation

|  | **PHQ-9** | | |  |
| --- | --- | --- | --- | --- |
|  | **OR** | **95% CI** | **p-value** |  |
| **Academic motivation profile** |  |  |  |  |
| Self-determined | REF |  |  |  |
| Non-self-determined | 1.79 | 1.32 – 2.44 | **<0.001** |  |
| **Demographic factors** |  |  |  |  |
| **Gender** |  |  |  |  |
| Male | REF |  |  |  |
| Female | 0.69 | 0.50 – 0.95 | **0.02** |  |
| **Ethnics** |  |  |  |  |
| Kinh | REF |  |  |  |
| Others | 1.03 | 0.52 – 2.04 | 0.94 |  |
| **Type of housemate** |  |  |  |  |
| Living alone | REF |  |  |  |
| Living with family | 0.78 | 0.48 – 1.24 | 0.29 |  |
| Living with friends | 0.91 | 0.59 – 1.40 | 0.67 |  |
| Others | 1.10 | 0.39 – 3.09 | 0.85 |  |
| **Marital status** |  |  |  |  |
| Single | REF |  |  |  |
| Married | 1.71 | 0.33 – 9.03 | 0.52 |  |
| Others | 3.25 | 0.84 – 12.66 | 0.09 |  |
| **Perceived financial burden** |  |  |  |  |
| Not having financial burden | REF |  |  |  |
| Having financial burden | 2.51 | 1.70 – 3.70 | **<0.001** |  |
| **Academic factors** |  |  |  |  |
| **Academic year** |  |  |  |  |
| First year | REF |  |  |  |
| Final year | 0.96 | 0.71 – 1.31 | 0.81 |  |
| **Academic major** |  |  |  |  |
| Doctor | REF |  |  |  |
| Bachelor | 0.90 | 0.60 – 1.35 | 0.61 |  |
| **Observations** | 1266 |  |  |  |
| *REF: Reference value*  *The bold p-value indicated statistical significance.* | | | | |

#

# eTable 14. Logistic Regression model on Academic Motivation

|  | **Self-determination index (SDI)** | | |  |
| --- | --- | --- | --- | --- |
|  | **OR** | **95% CI** | **p-value** |  |
| **Demographic factors** |  |  |  |  |
| **Gender** |  |  |  |  |
| Male | REF |  |  |  |
| Female | 0.84 | 0.66 – 1.06 | 0.14 |  |
| **Ethnic groups** |  |  |  |  |
| Kinh | REF |  |  |  |
| Others | 1.01 | 0.60 – 1.69 | 0.98 |  |
| **Type of housemate** |  |  |  |  |
| Living alone | REF |  |  |  |
| Living with family | 0.90 | 0.63 – 1.30 | 0.58 |  |
| Living with friends | 0.86 | 0.62 – 1.20 | 0.39 |  |
| Others | 0.67 | 0.28 – 1.60 | 0.36 |  |
| **Marital status** |  |  |  |  |
| Single | REF |  |  |  |
| Married | 0.68 | 0.19 – 2.46 | 0.56 |  |
| Others | 0.78 | 0.20 – 2.99 | 0.71 |  |
| **Perceived financial burden** |  |  |  |  |
| Not having financial burden | REF |  |  |  |
| Having financial burden | 1.02 | 0.74 – 1.41 | 0.90 |  |
| **Academic factors** |  |  |  |  |
| **Academic year** |  |  |  |  |
| First year | REF |  |  |  |
| Final year | 1.98 | 1.57 – 2.48 | **<0.001** |  |
| **Academic major** |  |  |  |  |
| Doctor | REF |  |  |  |
| Bachelor | 0.76 | 0.57 – 1.01 | 0.06 |  |
| **Observations** | 1723 |  |  |  |
| *REF: Reference value*  *The bold p-value indicated statistical significance.* | | | | |

# eTable 15. Logistic Regression model on Academic Motivation without multiple imputation

|  | **Self-determination index (SDI)** | | |  |
| --- | --- | --- | --- | --- |
|  | **OR** | **95% CI** | **p-value** |  |
| **Demographic factors** |  |  |  |  |
| **Gender** |  |  |  |  |
| Male | REF |  |  |  |
| Female | 0.85 | 0.66 – 1.09 | 0.19 |  |
| **Ethnic groups** |  |  |  |  |
| Kinh | REF |  |  |  |
| Others | 1.16 | 0.69 – 1.97 | 0.58 |  |
| **Type of housemate** |  |  |  |  |
| Living alone | REF |  |  |  |
| Living with family | 0.93 | 0.65 – 1.33 | 0.69 |  |
| Living with friends | 0.88 | 0.63 – 1.23 | 0.45 |  |
| Others | 0.65 | 0.27 – 1.57 | 0.34 |  |
| **Marital status** |  |  |  |  |
| Single | REF |  |  |  |
| Married | 0.58 | 0.15 – 2.28 | 0.43 |  |
| Others | 0.69 | 0.17 – 2.80 | 0.60 |  |
| **Perceived financial burden** |  |  |  |  |
| Not having financial burden | REF |  |  |  |
| Having financial burden | 1.09 | 0.77 – 1.54 | 0.64 |  |
| **Academic factors** |  |  |  |  |
| **Academic year** |  |  |  |  |
| First year | REF |  |  |  |
| Final year | 1.90 | 1.50 – 2.39 | **<0.001** |  |
| **Academic major** |  |  |  |  |
| Doctor | REF |  |  |  |
| Bachelor | 0.79 | 0.59 – 1.07 | 0.13 |  |
| **Observations** | 1293 |  |  |  |
| *REF: Reference value*  *The bold p-value indicated statistical significance.* | | | | |
